# Glomus tumor of the hyoid bone: a case report and literature review

**DOI:** 10.3389/fonc.2025.1512465

**Published:** 2025-04-04

**Authors:** Mingrui Shi, Ming Han, Jiale Wang, Qi Zhao, Chenghao Ren, Huan Li, Zihui Yang, Jianhua Wei, Xinjie Yang

**Affiliations:** ^1^ State Key Laboratory of Oral and Maxillofacial Reconstruction and Regeneration, National Clinical Research Center for Oral Diseases, Shaanxi Clinical Research Center for Oral Diseases, Department of Oral and Maxillofacial Surgery, School of Stomatology, The Fourth Military Medical University, Xi’an, China; ^2^ Department of Pathology, Xijin Hospital and School of Basic Medicine, The Fourth Military Medical University, Xi’an, China

**Keywords:** glomus tumor, hyoid bone, case report, literature review, differential diagnosis

## Abstract

Hyoid glomus tumors represent an exceptionally rare clinical entity. This study details a case presentation of a hyoid glomus tumor accompanied by a comprehensive systematic review, aiming to expand the clinical and pathological understanding of these uncommon neoplasms while evaluating therapeutic approaches. CT imaging revealed hyoid bone destruction with features suggestive of a borderline neoplasm. Histopathological examination demonstrated local spindle-shaped cells exhibiting a chicken claw-like morphology, which showed strong immunoreactivity for SMA, calponin, and collagen type IV - findings consistent with classical glomus tumor characteristics. The patient was ultimately diagnosed with a glomus tumor of uncertain malignant potential. Postoperative recovery proceeded favorably, with serial follow-up imaging studies demonstrating no evidence of recurrence or residual disease over several months of surveillance.

## Introduction

Glomus tumors (GTs) are perivascular mesenchymal neoplasms composed of modified smooth muscle cells, classified with myopericytoma, myofibroma, and angioleiomyoma. In 1812, Wood first described this disease as painful subcutaneous nodules, and Masson found that it originated from the normal glomus and named it GT in 1924. The general pathogenesis is the transformation of the arteriovenous anastomose-vascular sphere, which is believed to be formed by abnormal proliferation of the vascular sphere under the action of induction (such as trauma). It is more common in the distal limbs, sublingual and visceral organs and especially in the gastrointestinal tract, bones and mediastinum. Glomus tumors are rare, accounting for less than 2% of all benign soft tissue tumors (1). It is more common in adults aged 20 to 50 years, and half of them are aged 40 to 50 years. Subungual glomus tumors are more common in women (2), while glomus tumors outside the fingers are more common in men. It mainly occurs in the fingertip, and the treatment options are surgical resection and carbon dioxide laser treatment, which prevent relapse (3).

## Case

**Figure 1:**
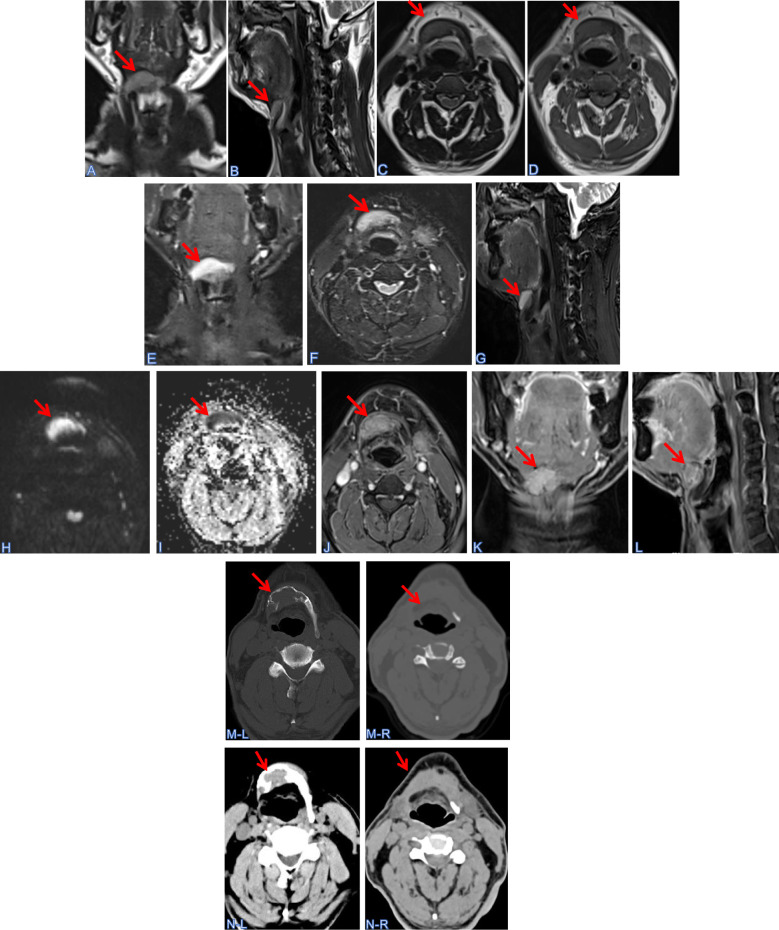
MR&CT imaging of head and neck masses. **(A-D)** Contrast-enhanced MR imaging: Expansive bone destruction of the hyoid bone to the right. **(A)** Coronal T2W1; **(B)** transverse T1W1; **(C)** sagittal T2W1; **(D)** transverse T2W1; **(E-G)** FS; **(H)** DWI; **(I)** ADC; **(J-L)** Multilocular expansive bone destruction of the hyoid bone. The tumor is approximately 3.3 cm×1.5 cm in size, with punctate calcification and a bone ridge. The bone cortex was discontinuous at the edge of the lesion, and soft tissue protrusion was observed. The lesions showed mild enhancement on contrast-enhanced scans. **(M)** Bone window in transverse axis view. **(N)** Soft window the transverse in axis position. (Left: before surgery; Right: 2 months after surgery).

A 60-year-old male with an 8-year history of hypertension (blood pressure maintained around 151/89 mmHg) presented with a right-sided neck mass persisting for over two weeks. Physical examination revealed asymmetrical hyoid bone enlargement (right > left) with a 3 cm firm, ill-defined, fixed mass located superior to the right hyoid body; the overlying skin remained intact with no tenderness. Comprehensive physical examination demonstrated no musculoskeletal deformities or skin tumors (including clinical appearance of neurofibromatosis type 1 (NF1)) and normal physiological reflexes. MRI revealed expansile right hyoid bone destruction (**Figures 1A–D**) showing hyperintense signal on fat-suppressed sequences with infiltrative margins. The lesion exhibited restricted diffusion (DWI hyperintensity with corresponding ADC hypointensity) indicating mylohyoid muscle involvement (**Figures 1E–I**), along with heterogeneous contrast enhancement (**Figures 1J–L**). Bilateral carotid sheath lymphadenopathy was noted (the largest node is about 2.1×1.0 cm) without cervical vertebral destruction. CT imaging (**Figures 1M, N**, the left panel) confirmed a multiloculated expansile hyoid lesion with right-sided predominance, containing punctate calcifications and osseous septations, while ultrasound identified an irregular 12×15×26 mm mass. These are imaging characteristics collectively suggested malignant etiology.

**Figure 2:**
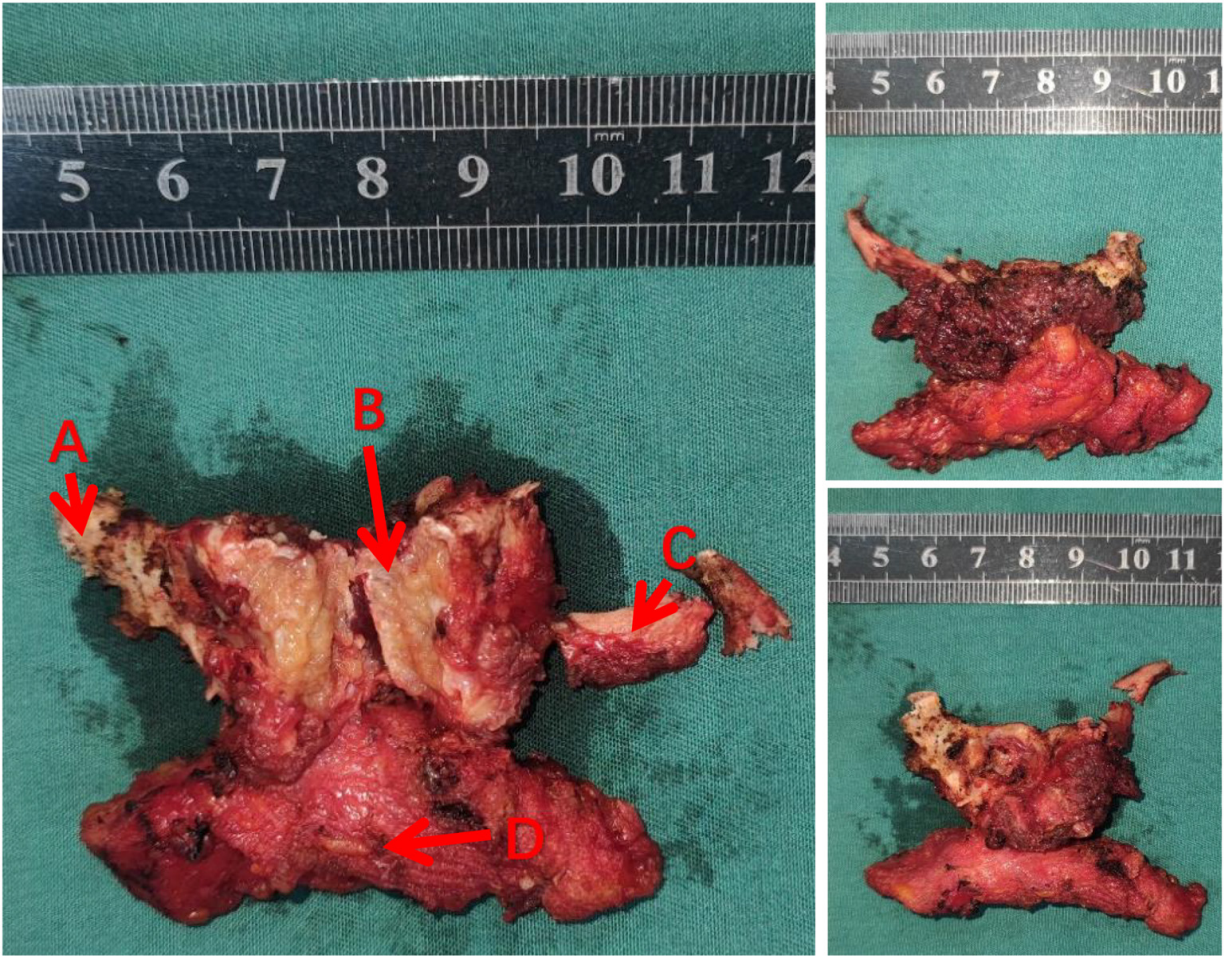
A photo of the tumor removed during surgery. **(A, C)** hyoid bone **(B)** tumors **(D)** lymphoid and connective tissue.

The mass was located within the hyoid bone with evident destruction and deep penetration. The central and right sides of the hyoid bone were notably affected. The mass, which was yellow–white with a soft texture and had a clear boundary and no obvious film, was resected and separated along the intact left side (**Figure 2**).

**Figure 3:**
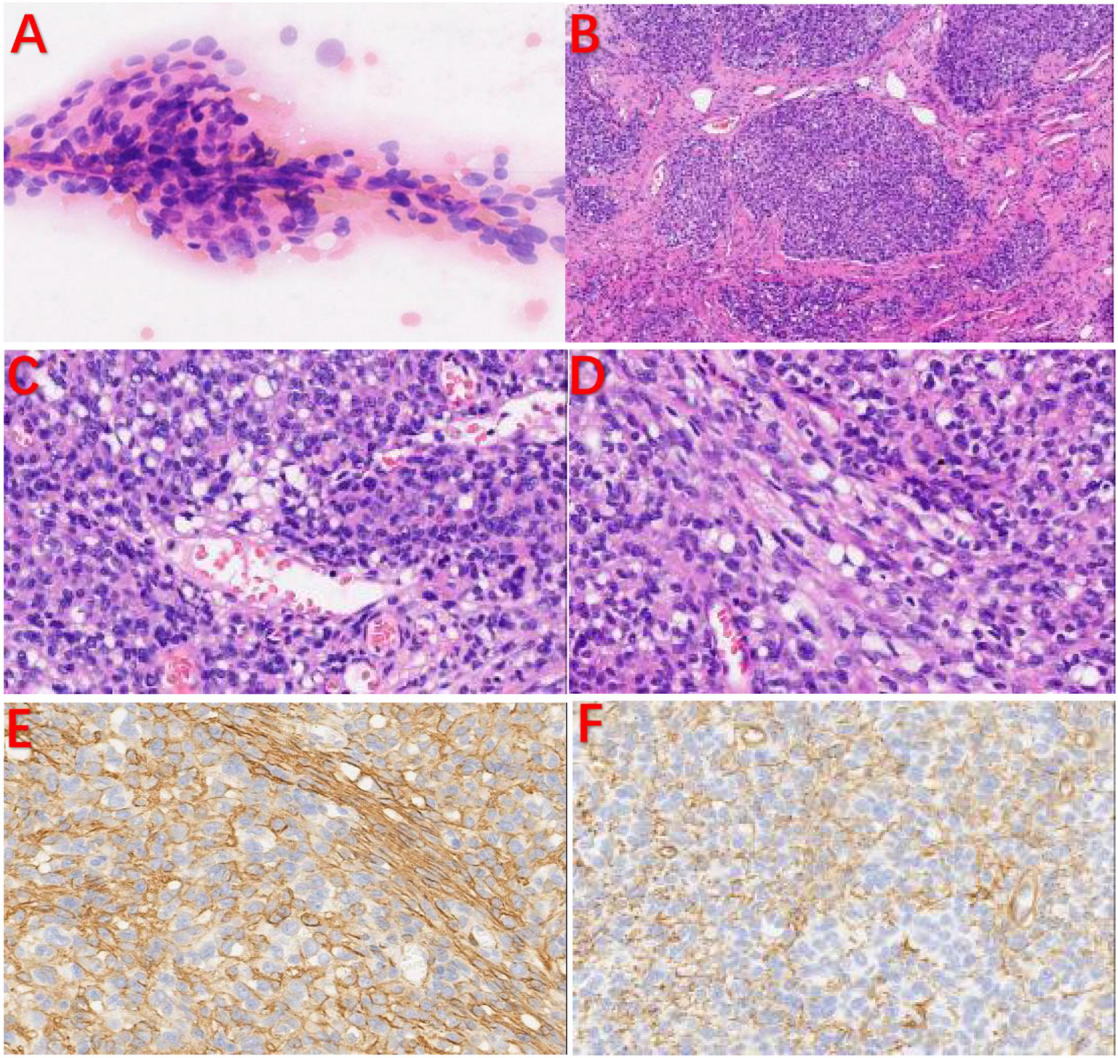
Pathological examination and IHC. **(A)** The tumor cells were distributed around blood vessels, and the cells were oval with fine chromatin. No nucleoli or mitotic figures were observed. **(B)** The tumor cells were lobulated, and the stroma was rich in blood vessels. **(C, D)** Some tumor cells were spindle-shaped. IHC: **(E, F)** The tumor cells were positive for SMA and type IV collagen in a chicken claw-like morphology.

Cytologically, the tumor cells were distributed around blood vessels, and the cells were oval with fine chromatin, and no nucleoli or mitotic figures were observed; these cells tended to be mesenchymal tumors (**Figure 3A**). Histologically, the tumor was well demarcated from the surrounding tissue and showed a lobulated growth pattern with a richly vascularized stroma. Mitotic figures were less than 2/10 HPF, and no pathological mitotic figures were observed (**Figures 3B–D**). The cells were positive for myogenic markers such as SMA, calponin, and type IV collagen (the latter of which showed a chicken claw-like morphology) while they were negative for Desmin, CD34, and S-100 (**Figures 3E, F**).

## Discussion

A literature review was conducted in Pubmed, CNKI, Medscape and other databases with the keywords “oral glomus tumor”, “tongue glomus tumor”, and “hyoid glomus tumor” from 1949 to 2024. A total of 45 relevant global cases were collected (**Table 1**). Male patients (n=25) were more susceptible than female patients (n=20). We found that 6 patients had tumors of the tongue (16.3%), 17 patients had tumors of the lip (37.8%), and 8 patients had tumors of the oral mucosa (17.8%). Other cases are mainly distributed in the jaw (4–45).

**Table 1:** Cases of oral glomus tumor reported in the global literature.

Author	Year	Age	Anatomical location	Clinical presentation	IHC	Follow-up time	Size(mm)	Outcome
Von Langer (4)	1949	52(M)	Hard palate	NA	NA	NA	NA	NA
King (5)	1954	32(M)	Gingiva	Tenderness	NA	NA	6	NA
Kirschner Strass-burg (6)	1962	56(M)	Gingiva/alveolar mucosa	NA	NA	NA	NA	NA
Grande and D’Angelo (7)	1962	42(M)	Hard palate	NA	NA	NA	NA	NA
Frankel (8)	1965	13(M)	buccal mucosa	NA	NA	NA	NA	NA
Harris and Griffin (9)	1965	35(F)	Periodontal/gum	Pain	NA	2 years	5*2.5	NED
Sidhu and Subherwal (10)	1967	10(F)	Hard palate	NA	NA	NA	NA	NA
Charles (11)	1976	17(F)	Hard palate	No	NA	NA	NA	NA
Lele (12)	1977	35(F)	Hard palate	Bleeding	NA	6 months	15*10	NED
Sato et al. (13)	1979	29(M)	Tongue	No	NA	NA	3	NA
Tajima et al. (14)	1981	63(F)	Tongue	No	NA	NA	NA	NA
Saku et al. (15)	1985	45(M)	Buccal mucosa	No	Actin(+)smooth muscle myosin(+)	NA	45*30*35	NA
Ficarra et al. (16)	1986	51(F)	Upper lip	No	NA	NA	20	NA
Moody et al. (17)	1986	65(F)	Upper lip	No	NA	NA	10*5*5	NA
Stajcic and Bojic (18)	1987	55(M)	Tongue	NA	NA	NA	NA	NA
Tokiwa et al. (19)	1990	36(M)	Gingiva	NA	NA	NA	NA	NA
Geraghty et al. (20)	1992	71(M)	Hard palate	No	NA	NA	15	NA
Kusama et al. (21)	1995	57(M)	Upper lip	Tenderness	S100(+), actin(+), desmin(+),vimentin (+), factor VIII(−)	4 years	NA	NED
Savaci et al. (22)	1996	55 (F)	Mucosa of mouth	Pain	NA	NA	10	NA
Sakashita et al. (23)	1997	54(M)	Upper lip	No	NA	NA	12	NA
Yu et al. (24)	2000	54(F)	Left mandibular region, lip, mucous membrane	No	smooth muscle actin(+), S-100(−)	NA	NA	NA
Kessaris et al. (25)	2001	46(F)	Hard palate	No	Vimentin (+), smooth muscle actin(+), actin(−), desmin(−) chromogranin(−), neuron-specific enolase(−), epithelial membrane antigen(−) cyto-keratin(−),factor VIII(−)	3 years	18	NED
Rallis et al. (26)	2004	85(F)	Upper lip	Pain	smooth muscle actin(+), muscle specific actin(+), vimentin (+), desmin(−),S-100(−), epithelial membrane antigen(−),neuron-specific enolase(−)AE1/3(−), Leu7(−), CD3,CD31,CD34,CD45,CD20(−), cytokeratin(−)	1.5 years	13*10*10	NED
Quesada R et al. (27)	2004	61(M)	Tongue	No	NA	7 years	30	Recurrence
Lanza et al. (28)	2005	65(M)	Lower lip	NA	NA	NA	NA	NA
Maeda et al. (29)	2005	20(M)	Jaw		Vimentin (+), smooth muscle actin(+), HHF35(+) keratin(−) S-100(−) factor VIII(−), desmin(−)	NA	NA	NA
Ide et al. (30)	2008	57(M)	Upper lip	NA	NA	NA	8	NA
Ide et al. (30)	2008	54(M)	Upper lip	NA	NA	NA	12	NA
Wang et al. (31)	2008	58(F)	Buccal mucosa	NA	NA	NA	NA	NA
Boros et al. (32)	2010	34(M)	Lower lip	No	smooth muscle actin(+), muscle specific actin(+), S-100(+), kerarin(−), epithelial membrane antigen(−),CD34(−), CD31(−), chromogranin(−)	5 years	15*15*11	NED
Yoruk et al. (33)	2010	30(F)	Buccal mucosa	No	smooth muscle actin(+),S-100(−), kerarin(−),p53(+) bcl2 (–)CD34(+),CD117(−)CD31(+), chromogranin(−)desmin(−) AE1/3(−)	1 years	20*11*5	NED
Derand III et al. (34)	2010	11(F)	Lower lip	No	pancytokeratin(−), vimentin (+), smooth muscle actin(+), S-100, factor VIII(−)	7 years	3	NED
Veros et al. (35)	2012	24(F)	Buccal mucosa	No	NA	2 years	10*10	Recurrence
Chou et al. (36)	2015	39(M)	Upper lip	NA	NA	NA	NA	NA
Kazuto et al. (37)	2016	44(M)	lower jawbone	Dull pain	Vimentin (+), muscle specific actin/HHF35(+), calponin (+), typeIV collagen (+), smooth- muscle-actin(−), cytokeratin(AE1/AE3)(−), cytokeratin(CAM5.2)(−), CK19(−), CD31(−), CD34(−), CD68(−), p63(−), S-100(−), factor VIII(−), desmin(−)	10 years	45*30*30	NED
Monaghan (38)	2017	73(M)	Upper lip	No	NA	NA	10	NA
Vasconcelos et al. (39)	2018	67 (F)	Upper lip mucosa	Pain	CD34(+), smooth-muscle-actin(+) Vimentin (+) S-100(−) cytokeratin(−)STAT-6 (–)	3.3 years	10	NED
Smith et al. (40)	2018	26(M)	Lower lip	Pain	HHF-35(+)SMA(+) AE1/3 (–) CD31、CD34 (–)	NA	15*5*5	NA
Smith et al. (40)	2018	58(F)	Tongue	No	SMA(+), MSA/HHF35(+)S100 (–)p63 (–)GFAP (–)AE1/3 (–)CD31、CD34(+)	1 months	20*10	NED
Zou et al. (41)	2018	24(F)	Mouth floor	Pain	VIM(+)αSMA(+)AE1 (–)AE3 (–)CD31 (–)CD34 (–) S-100 (–) Ki67 (+, 5%)	4 years	28*18*21	NED
Sánchez-Romero C et al. (42)	2019	51 (F)	Upper lip mucosa	Pain	VIM(+)CD34(+),αSMA(+)HHF35(+) hCaldesmon(+)AE1/AE3(+)S-100(+)desmin(+)	NA	10	NA
Naji Rad S et al. (43)	2020	62(M)	Lower lip mucosa	No	NA	1 year	10	NED
Chandran S et al. (44)	2022	8(F)	Lowerjawbone	Pain	Vimentin (+)SMA (+)desmin (–)p63 (–)CD34 (–)CD45 (–)	NA	20*45*20	NA
Afroozi B (45)	2023	37(M)	Buccal mucosa	No	CD34(+)AE1/3 (–)S100 (–)vimentin(+)SMA(+)CD31 (–) p63 (–)	2 years	20*20	NED
Our case	2023	60(M)	Tongue bone	No	SMA(+),CD56(+),Hcald(+),Calponin (+) Collagen IV(+) Desmin (–)CK(AE1/AE3) (–), EMA (–), CD34 (–), S100 (–), Syn (–), CgA (–), Ki-67 (+,1%)	NA	12*15*26	NED

NA, not available; NED, no evidence of disease.

GT usually presents as a solitary small red–blue nodule with obvious pain when cold and touch clinically. Approximately 10% of patients have multiple lesions, and 9% to 60% of patients have abnormal bone changes. GT in the oral cavity is rare, with an incidence of only 0.6% (32). Approximately 45 patients were identified, with a wide age of onset (8 to 85), a mean age of 45 years, and more common males. GT in the bone is most common in the phalanx, followed by the vertebral body. Imaging shows osteolytic changes with sclerotic edges, which should be differentiated from bone hemangioma, aneurysmal bone cyst, bone metastasis cancer and tuberculosis, etc (46).

The tumor cells were small, round, uniform in size, lightly eosinophilic with occasional eosinophilic or epithelioid cell morphology, hyalinization or a mucinous matrix but showed no necrosis. IHC revealed positivity for SMA, Syn, and collagen IV, while S-100 was positive. However, CK, desmin, and CD34 tested negative. A recent study revealed that *BRAF* V600E mutations may be associated with a malignant phenotype in glomus tumors (47); however, larger cohorts and multicenter studies are required to confirm these findings.

### Differential diagnosis

(1) Myopericytoma: There are no uniform round cells, and the characteristic oval and spindle cells grow around the blood vessels. There was some overlap with the morphology of the glomus tumor.
(2) Paragangliomas: These tumors exhibit nested organ-like growth. IHC: SYN(+), CgA(+), S-100(+), and SMA(+).
(3) Angioleiomyoma is composed of mature smooth muscle cells arranged in fascicles lacking round cells of uniform size. IHC: SMA and Desmin(+).
(4) Neuroendocrine tumors: Tumor cells with speckly chromatin in the nucleus. IHC revealed CK, SYN and CgA (+) SMA (+) and Syn(+) when they occurred in the gastrointestinal tract, and these tumors were easily misdiagnosed as neuroendocrine tumors.
(5) Suquet-Hoyer: This structure appears as a narrow lumen lined by a single layer of endothelial cells and surrounded by 4 to 6 layers of spheroid cells, which are regarded as specialized smooth muscle cells. Sometimes, this normal structure is observed in specimens from distal limb biopsies performed for other reasons and is mistaken for GT (48).
(6) Aneurysmal bone cyst: CT clearly revealed peritumoral ossification and calcification. The MR plain scan signal was heterogeneous; the fluid–fluid level in the lesion is its characteristic manifestation on MR images. An enhanced scan revealed uneven progressive enhancement (49). Eccentric balloon-like expansion may be observed on X-ray, and a large amount of blood can be drawn by local puncture.
(7) Hyoid chondroma: the tumor is located in the upper neck of the hyoid bone plane, is surrounded by a hyoid muscle group and is imperceptible, and can slowly occur in the mouth. Subjective symptoms are not obvious and are not easy to detect early. The mass is generally hard, well-defined, and benign and moves with the hyoid bone when swallowing (50).
(8) Hyoid chondrosarcoma: This type of chondrosarcoma is overwhelmingly low grade and presents as a slow-growing, painless mass on the lateral side of the neck. CT shows a dilated tumor with cortical destruction and matrix calcification, and focal exophytic lesions with intimal sector features can be seen in rapidly progressing chondrosarcomas (51). T1lWI is low, T2WI shows peripheral enhancement, and T8WI is high (52).
(9) Radiation-induced osteonecrosis of the hyoid bone: This is a common complication after radiotherapy for tumors that are often misdiagnosed as recurrent tumors. The typical imaging manifestations are cortical fragmentation, bone fragmentation, and air filling in the bone. Some patients have soft tissue enhancement signals on PET/CT, suggesting that FDG activity is significantly enhanced and is easily mistaken for tumor recurrence (53).
(10) Thyrohyoid cysts: They are most common near the hyoid bone (54). Ultrasound revealed a clear boundary, regular shape, and clear fluid inside. In some cases, strip-like septa can be seen. When the course of disease is long or complicated with infection, the internal echo increases, and the floating light spot can be seen the same for the echo of a solid mass, but the posterior echo is enhanced (55). The hyoid bone is rare, and inactive thyroid tissue and cholesterol particles can be found in the cyst wall (56).

GT often occurs in the glomus cell-rich parts of the extremities, especially under the nail bed of the fingers and toenails, and rarely in the skin, bone or internal organs. Lingual GTs are mostly located on the back of the tongue and are rarely more than 1 cm long and have red or medium textures and clear boundaries, without the triad of subungual GTs (pain, tenderness, cold shock) (57). According to the 2013 WHO soft tissue classification criteria, the diagnostic criteria for malignant glomus tumors are (1) marked nuclear atypia and any level of mitotic figures or (2) the presence of atypical mitotic figures. When the histological appearance of the tumor does not meet the above criteria for the diagnosis of malignancy but there is at least one atypical feature (e.g. a diameter greater than 2 cm, increased mitotic count, deep location, etc.) should be called a “glomus tumor of uncertain malignant potential” (GT-UMP) (58). According to the size and location of the tumor, this patient was diagnosed with GT-UMP. As for the IHC, the tumor cells were positive for α-SMA, MSA, h-caldesmon, calponin, vimentin and collagen IV. CD34 was positive in some patients, but desmin, AE1/AE3 and S100 were negative.

At present, the most common and effective method for treating GT is surgical local resection, but there is still a possibility of recurrence. For laser treatment, a C02 laser with an output power of 2~3 W can be used to punch into the subcutaneous or nail bed for direct coagulation, or an ND: YAG laser with an output power of 3~5 W and fiber inserted directly into the lesion for coagulation can be used. The treatment is simple and easy, and no special postoperative care is needed.

Typically glomus tumors are benign, but malignant glomus tumors have high potential for recurrence and metastasis. The prognosis of patients with malignant glomus tumors is good. However, the number of follow-up cases in the literature is limited, and the follow-up time is short, so the follow-up should be strengthened in practical work.

## Data Availability

The original contributions presented in the study are included in the article/supplementary material. Further inquiries can be directed to the corresponding author.
